# Relationship between Levels of Pre-Stroke Physical Activity and Post-Stroke Serum Insulin-Like Growth Factor I

**DOI:** 10.3390/biomedicines8030052

**Published:** 2020-03-04

**Authors:** N. David Åberg, Gustaf Gadd, Daniel Åberg, Peter Hällgren, Christian Blomstrand, Katarina Jood, Michael Nilsson, Fredrick R. Walker, Johan Svensson, Christina Jern, Jörgen Isgaard

**Affiliations:** 1 Department of Internal Medicine, Institute of Medicine, The Sahlgrenska Academy, University of Gothenburg, SE-41345 Gothenburg, Sweden; gustaf.gadd@vgregion.se (G.G.); daniel.aberg@medic.gu.se (D.Å.); johan.svensson@medic.gu.se (J.S.); jorgen.isgaard@medic.gu.se (J.I.); 2 Region Västra Götaland, Sahlgrenska University Hospital, Gothenburg SE-41345, Sweden; peter.hallgren@vgregion.se; 3 Department for Clinical Neuroscience, Institute of Neuroscience and Physiology, The Sahlgrenska Academy, University of Gothenburg, SE-405 30 Gothenburg, Sweden; christian.blomstrand@neuro.gu.se (C.B.); katarina.jood@neuro.gu.se (K.J.); 4 School of Biomedical Sciences and Pharmacy and the Priority Research Centre for Stroke and Brain Injury, the University of Newcastle, University Dr, Callaghan, NSW 2308, Australia; michael.nilsson@newcastle.edu.au (M.N.); rohan.walker@newcastle.edu.au (F.R.W.); 5 Hunter Medical Research Institute, Lot 1, Kookaburra Cct, New Lambton Heights, NSW 2305, Australia; 6 Department of Laboratory Medicine, Institute of Biomedicine, The Sahlgrenska Academy, University of Gothenburg, SE-41345 Gothenburg, Sweden; christina.jern@neuro.gu.se; 7 Department of Clinical genetics and genomics, Sahlgrenska University Hospital, SE-41345 Gothenburg, Sweden

**Keywords:** stroke, cerebrovascular disease, insulin-like growth factor I, ischemic stroke, stroke severity, physical activity

## Abstract

Physical activity (PA) and insulin-like growth factor I (IGF-I) have beneficial effects for patients who have suffered an ischemic stroke (stroke). However, the relationship between the levels of PA and IGF-I after stroke has not been explored in detail. We investigated the pre-stroke PA level in relation to the post-stroke serum IGF-I (s-IGF-I) level, at baseline and at 3 months after the index stroke, and calculated the change that occurred between these two time-points (ΔIGF-I). Patients (*N* = 380; 63.4% males; mean age, 54.7 years) with data on 1-year leisure-time pre-stroke PA and post-stroke s-IGF-I levels were included from the Sahlgrenska Academy Study on Ischemic Stroke (SAHLSIS). Stroke severity was assessed using the National Institutes of Health Stroke Scale (NIHSS). Pre-stroke, leisure-time PA was self-reported as PA1–4, with PA1 representing sedentary and PA2–4 indicating progressively higher PA levels. Associations between s-IGF-I and PA were evaluated by multiple linear regressions with PA1 as the reference and adjustments being made for sex, age, history of previous stroke or myocardial infarctions, cardiovascular risk factors, and stroke severity. PA correlated with baseline s-IGF-I and ΔIGF-I, but not with the 3-month s-IGF-I. In the linear regressions, there were corresponding associations that remained as a tendency (baseline s-IGF-I, *p* = 0.06) or as a significant effect (ΔIGF-I, *p* = 0.03) after all the adjustments. Specifically, for each unit of PA, ΔIGF-I increased by 9.7 (95% CI 1,1−18.4) ng/mL after full adjustment. This supports the notion that pre-stroke PA is independently related to ΔIGF-I.

## 1. Introduction

Stroke is a condition that is associated with high costs for the healthcare system, with the financial burden and suffering being significantly higher in cases of moderate and severe stroke than in mild cases [1]. To improve stroke prevention, knowledge of key risk factors, especially those that are modifiable, such as physical activity (PA), is essential. Insulin-like growth factor I (IGF-I) has neuroprotective effects and promotes brain plasticity in a positive fashion after brain injuries [2]. In line with this, higher serum insulin-like growth factor I (s-IGF-I) levels are associated with improved recovery after ischemic stroke, hereinafter referred to as stroke [3,4,5,6,7]. Moreover, a low level of s-IGF-I is a risk factor for the occurrence of stroke [8]. The expression of s-IGF-I is upregulated by growth hormone (GH) from the pituitary, although IGF-I is also related to other factors, showing a negative relationship with age and insulin resistance and a positive relationship with PA [9,10].

Studies that have investigated the relationship between stroke and s-IGF-I have produced variable findings of increased [5,11], unchanged [6], and decreased post-stroke levels of s-IGF-I [3,12]. It appears that this variability is largely attributable to differently matched controls (or no matching at all) at the time of the post-stroke sampling or to whether there was an intra-individual collection of serial serum samples. Data from our group and others indicate that there is at least some increase in the level of s-IGF-I during the first days after stroke [5,11], whereas the s-IGF levels are lower 3 months later [5]. As PA [13,14] and s-IGF-I [3,4,5,6,7] each have beneficial effects following stroke and their mutual relationship after stroke has been scarcely explored, the s-IGF-I response with respect to pre-stroke PA may be of relevance to recovery after brain injuries. The notion that PA is related to circulating IGF-I in terms of stroke is also underlined by experimental data showing that exercise-induced recovery after a chemical insult to the brain is attenuated by the administration of anti-IGF-I antibody-containing serum [15]. Furthermore, it appears that not only the s-IGF-I level per se but also the dynamic post-stroke decrease in s-IGF-I is able to predict stroke outcome [7]. 

The reports linking s-IGF-I to exercise and PA levels, including the situation of post-injury brain recovery after a chemical insult, prompted us to investigate the self-reported pre-stroke PA levels and post-stroke s-IGF-I responses in patients with stroke. In the present study, we investigated the potential associations between pre-stroke PA and post-stroke s-IGF-I, at baseline (baseline s-IGF-I) and at 3 months post-stroke (3-month s-IGF-I), and including the change in s-IGF-I calculated between these two time-points (ΔIGF-I), and we examined whether these associations were retained in different multiple linear regression models. 

## 2. Results

### Pre-stroke Physical Activity and Post-stroke s-IGF-I

**Figure 1:**
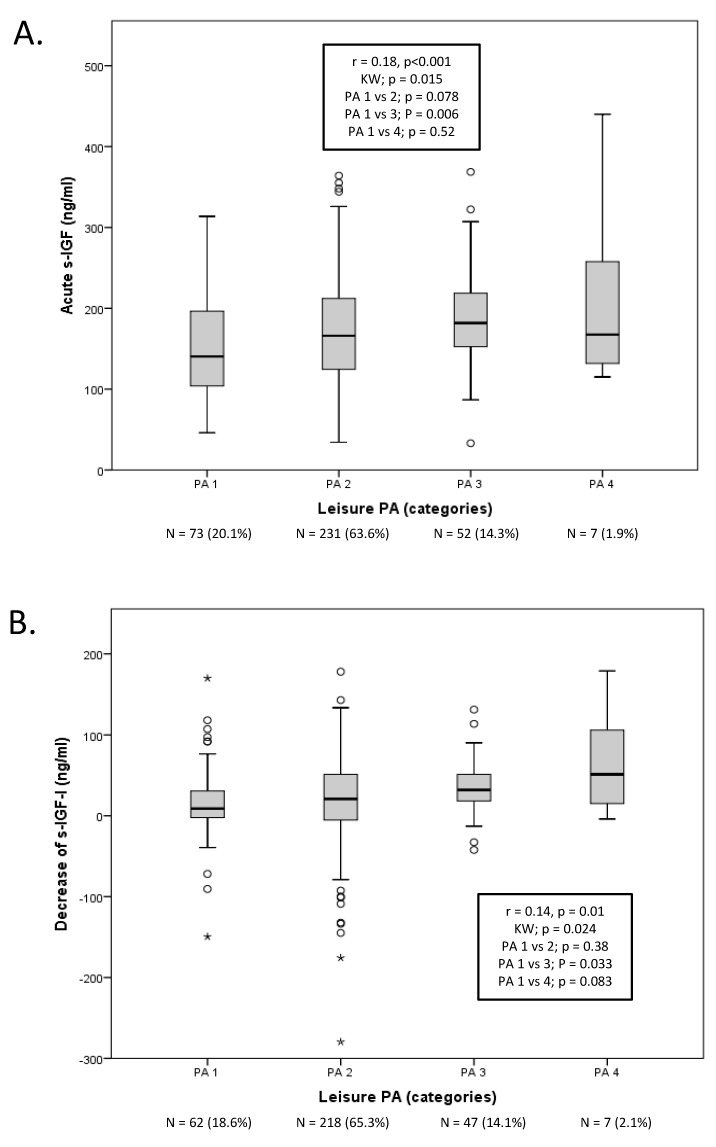
Serum insulin-like growth factor I insulin-like growth factor I (IGF-I) (ng/mL) according to pre-stroke physical activity (PA) levels 1-4, as assessed by Salting-Grimby. (**A**). Baseline IGF-I according to pre-stroke PA. (**B**). ΔIGF-I, according to pre-stroke PA. ΔIGF-I represents the change (decrease) in serum IGF-I (s-IGF-I) from baseline to 3 months post-stroke. Thus, a greater positive value in the figure represents a greater decrease. Box plots showing the median, interquartile ranges, whiskers, outliers (circles) and extremes (asterisks, see methods). The results of the Spearman correlation are given in the text box (rho, *r*, and *p*-values). The Kruskal–Wallis (KW) overall *p*-value is given as well as *p*-values for the Bonferroni-corrected (for 3 comparisons) comparisons vs. PA1. For baseline s-IGF-I, there were *N* = 363 (95.5%), and for ΔIGF-I, there were *N* = 334 (87.8%), with numbers (*N*) and relative percentages (%) for each PA1–4 category given in the Figure.

The baseline characteristics of the 380 patients included in this study are summarized in Table 1. The cardiovascular risk factors of diabetes, hypertension, and smoking were commonly observed in this cohort, as before [5,7,16,17]. The pre-stroke PA level of the studied cohort was skewed towards a lower level of PA, with PA1 and PA2 constituting 82.9% of the whole cohort and with very few of the participants being in the PA4 group, as shown in Table 1. Stroke severity (baseline NIHSS) was not correlated with pre-stroke PA. Significant crude negative correlations between PA and body mass index (BMI), diabetes, and smoking were noted (Table 1). However, although there was a significant relation between s-IGF-I and PA (Figure 1), none of the IGF-I variables (baseline s-IGF-I, 3-month s-IGF-I, or ΔIGF-I) correlated with BMI (*r* = −0.034, *p* = 0.52; *r* = 0.016, *p* = 0.77; *r* = −0.029, *p* = 0.59, respectively).

The median baseline level of s-IGF-I was 164 ng/mL, the median ΔIGF-I was 21.3 ng/mL, and the median 3-month s-IGF-I was 147 ng/mL. As compared to the PA1 group, the baseline s-IGF-I was significantly higher in the PA2 (19%) and PA3 (30%) groups, and ΔIGF-I was significantly higher in the PA3 (260%) and PA4 (474%) groups (Figure 1). There were no statistically significant differences in the 3-month s-IGF-I level between the different PA levels (not shown). However, there were statistically significant correlations between PA and the baseline s-IGF-I and ΔIGF-I (Figure 1), but not with the 3-month s-IGF-I (*r* = 0.092, *p* = 0.084).

The significant correlations between PA and s-IGF-I (baseline s-IGF-I and ΔIGF-I) were further assessed by multiple linear regression analyses, as shown in Table 2. In Models 1 and 2, with adjustments made for age, sex, and history of previous stroke or myocardial infarctions, the association between baseline s-IGF-I and PA remained significant, whereas only a tendency towards an association remained after additional adjustments were made for cardiovascular risk factors. The association between ΔIGF-I and PA, however, remained significant in all of the models (Model 4, *p* = 0.028). Specifically, with full adjustment (Model 4), for each unit of PA, ΔIGF-I increased by 9.7 (95% CI 1,1−18.4) ng/mL.

## 3. Discussion

### 3.1. Physical Activity and s-IGF-I

To the best of our knowledge, this is the first study to demonstrate that PA prior to ischemic stroke is not only associated with the baseline s-IGF-I but also with the magnitude of the decrease in s-IGF-I (ΔIGF-I) during the first 3 months after the ischemic event. The associations remained significant in all the multiple regression models for ΔIGF-I, as opposed to the attenuated association for PA and baseline s-IGF-I. The finding that the 3-month IGF-I level did not differ significantly between the PA groups implies that post-stroke IGF-I represents an acute response to the ischemic stroke, mainly in terms of the baseline s-IGF-I and ΔIGF-I values.

Two major discoveries emerge from the present study: 1) the pre-stroke PA association with post-stroke baseline s-IGF-I and ΔIGF-I shows different attenuations, with only the first association being attenuated (to a tendency) after adjustments for cardiovascular covariates. This may reflect mediation or confounding, although the data within our study do not allow us to discriminate between these two possibilities. Notwithstanding uncertain causality, it appears that post-stroke s-IGF-I is in some way related to PA. In light of the present results, it is noteworthy that ΔIGF-I has previously been shown to have the strongest association with the beneficial effects on stroke outcome [7]. This may reflect the importance of the response of IGF-I to the acute ischemic event. In particular, decreasing levels in IGF-I, i.e., ΔIGF-I, post-stroke appears to be associated with good functional outcome after ischemic stroke [7,11]. In animal experiments, the degree of physical exercise has been shown to be positively associated with the uptake of circulating IGF-I into the brain in a situation with an injury caused by a chemical insult [15], which has some similarities to stroke. Although this is an observational study without assessment of IGF-I uptake into the brain, the present results are in line with those previous results and support the notion that a high level of PA facilitates high-level uptake of peripheral s-IGF-I, which may be reflected in the rather robust association with ΔIGF-I. In this aspect, it is of interest to mention that previously we have shown that ΔIGF-I is associated with the most favorable outcome after stroke [7]. Finally, it should be noted that although the causal relationship between PA and s-IGF has been debated [10] and been considered complex [18], an association between s-IGF-I and aerobic fitness and muscular endurance has been demonstrated in healthy individuals [19]. This is also in line with a report showing that the estimated pre-stroke aerobic capacity is crudely correlated with post-stroke s-IGF-I assessed 0–72 h after the stroke [20], although neither the PA levels per se nor any regressions were analyzed. Furthermore, while several studies have determined that PA prior to stroke is associated with improved outcomes [13,21,22], this is one of the first studies to implicate IGF-I as a contributing mechanism. Collectively, these findings suggest that greater consideration should be given to tracking the serum levels of IGF-1 in recovering patients.

### 3.2. Strengths and Limitations

This is a prospective study with dual sample points for the biomarker of interest, IGF-I. Compared with previous studies that have looked at changes in post-stroke IGF-I, the number of patients in the Sahlgrenska Academy Study on Ischemic Stroke (SAHLSIS) cohort is relatively high [11]. However, since the PA levels were skewed towards lower values, there was only a low number of patients with PA4 (*N* = 8), weakening the specific analysis of the effect in that group. Another weakness is the use of self-reported retrospective data collected shortly after an acute stroke, although the Saltin-Grimby Physical Activity Level Scale (SGPALS) is a well-established scale for assessing the history of PA [21,23]. Specifically, in the present study, the level of PA was taken as the self-reported average during the 12 months preceding the index ischemic stroke. Finally, it would be of great interest to have data on post-stroke PA levels and pre-stroke s-IGF-I. However, to investigate the latter, a completely different study design would be required (such as a long-term observational study of a healthy cohort). 

## 4. Materials and Methods 

### 4.1. Subjects and Design

The design of SAHLSIS has been described previously [5,16]. Briefly, adult patients aged <70 years with first-ever or recurrent acute IS were recruited consecutively at four Stroke Units in western Sweden in the period of 1998–2003. However, in the present study, only patients from the Stroke Unit at the Sahlgrenska University Hospital were included, as analyzed previously (*N* = 407) [5]. In addition, in this study, we used data from the patients regarding pre-stroke PA and s-IGF-I for any of the time-points of baseline or 3 months post-stroke (*N* = 380) (Table 1). At inclusion into SAHLSIS, the pre-stroke PA level for the last 12 months before the index stroke was assessed in a questionnaire using a self-report scale, with four levels of PA, the SGPALS, in which PA1 represents sedentary, and PA2 to PA4 representing progressively higher levels of PA [23]. In short, PA2 represents an “active group”, PA3 an “intensive group”, and PA4, an “elite intensive group”; a more detailed presentation is given in the Supplementary Material.

The s-IGF-I was measured in the acute phase (i.e., early post-stroke, baseline s-IGF-I; at a median of 4 days post-stroke) and at 3 months after the index stroke (3-month s-IGF-I) [5] (see also Supplementary Material, which includes data on variation and detection limits). The change (decrease) in s-IGF-I between baseline and 3 months, ΔIGF-I, was calculated [7]. Data on sex, age, history of previous stroke and/or myocardial infarctions/peripheral artery disease, body mass index (BMI), conventional vascular risk factors [presence of hypertension, diabetes mellitus, smoking, and concentration of low-density lipoprotein (LDL), mmol/L], and initial stroke severity were recorded and included in the analyses. The severity of stroke in the acute phase was initially assessed according to the Scandinavian Stroke Scale (SSS), expressed as the maximum score during days 0–10, and this was transformed into the more frequently used NIHSS according to the validated algorithm of NIHSS = 25.68 − 0.43 × SSS [24]. BMI, hypertension, diabetes, and smoking were defined as described previously [5,7,16,17], and functional outcome 3 months and 2 years after IS was assessed using the modified Rankin Scale (mRS); all of these details are further described in Supplementary Materials. Written informed consent was obtained from the patient or next of kin. This study was approved by the Ethics Committee of the University of Gothenburg (#Ö469-99, 03/02/2000). 

### 4.2. Statistical Analysis

Continuous data are presented as median values and interquartile ranges (IQR) due to the non-parametric distribution. Box-plots are given according to the Tukey distribution; medians, 25th–75th percentiles, and ranges (whiskers) are all defined as values between the 25th percentile − [1.5 × interquartile range (IQR)] and the 75th percentile + (1.5 × IQR), with outliers (circles) and extremes (asterisks). The Shapiro–Wilk test was performed for testing normality. The Kruskal–Wallis H-test, followed by a series of pairwise Bonferroni-corrected Mann–Whitney *U*-tests, was performed. Spearman correlation was used for testing correlations between PA and the various parameters (Table 1 and Table 2). Multiple linear regression was used for determining unstandardized B-values (equivalent to s-IGF-I ng/mL at baseline or as the change in s-IGF-I level at 3 months, ΔIGF-I) and 95% confidence interval (CI) per unit of pre-stroke PA level. Adjustments were made for sex (S), age (A), history of stroke or myocardial infarction (history), BMI (B), conventional vascular risk factors (hypertension, diabetes, smoking, LDL) (C), and initial stroke severity (I). For LDL, which had the most missing values, imputation was used to replace the missing values with the mean LDL value. The statistical significance level was set at a two-tailed *p*-value < 0.05. Statistical analyses were performed using the IBM^®^ SPSS^®^ ver. 25 software (SPSS Inc., Chicago, IL, USA).

## 5. Conclusions

The present study reveals an independent association between pre-stroke PA and post-stroke s-IGF-I. Of the IGF-I variables studied, the change in level between the acute phase and 3 months post-stroke (ΔIGF-I) showed the strongest association with PA, and this association was retained in all the regression models. Further studies are needed to uncover whether the pre-stroke PA results in a priming effect that exerts beneficial effects with respect to the functional outcome or whether this is an effect of continuous post-stroke PA in those persons who were already physically active prior to the index stroke. 

## Figures and Tables

**Table 1:** Baseline parameters and crude correlations vs. physical activity (PA).

Parameter	Value	Subjects	Missing	Correlation (vs. PA)	
		N	N (%)	*r*	*p*
Patients with stroke (N)	380	380	0 (0)	N/A	N/A
Age, years, median (IQR)	57.0 (49.8–63.0)	380	0 (0)	−0.07	0.13
Male/female, N (male %)	241/139 (63.4)	380	0 (0)	−0.091	0.078
History of stroke, N (%)	71 (18.6)	380	0 (0)	−0.152	**0.006**
History of cardiovascular disease, N (%)	117 (30.7)	380	0 (0)	−0.018	0.75
BMI, kg/m^2^, median (IQR)	25.7 (23.6–28.7)	377	3 (0.8)	−0.122	**0.018**
Diabetes, N (%)	70 (18.4)	380	0 (0)	−0.179	**<0.001**
Hypertension, N (%)	206 (54.2)	376	4 (1.1)	−0.051	0.32
Current smoking, N (%)	147 (38.7)	380	0 (0)	−0.164	**0.001**
LDL, mmol/L, median (IQR)	3.3 (2.6–4.0)	342	38 (10)	−0.015	0.79
Imputed LDL, mmol/L, median (IQR)	3.3 (2.7–3.9)	380	0 (0)	−0.016	0.76
3-month mRS, median (IQR)	2 (1–2)	359	21 (5.5)	−0.029	0.59
PA, median (IQR)	2 (2–2)	380	0 (0)	N/A	N/A
PA 1, N (%)	3.54 (1.6–9.56)	76 (20.0)	0 (0)	N/A	N/A
PA 2, N (%)	2.46 (0.74–6.33)	239 (62.9)	0 (0)	N/A	N/A
PA 3, N (%)	2.46 (1.6–10.2)	57 (15.0)	0 (0)	N/A	N/A
PA 4, N (%)	2.46 (1.82–12.35)	8 (2.1)	0 (0)	N/A	N/A
Stroke severity, NIHSS, median (IQR)	2.89 (1.17–7.62)	380	0 (0)	−0.017	0.75

Footnotes: The numbers of subjects (*N*) and percentages (%) are given relative to the whole cohort (*N* = 380). *p*-values < 0.05 are indicated in bold. Crude correlations, according to the method of Spearman (rho values, *r*; and *p*-values, *p*), are shown for each parameter vs. PA. History of cardiovascular disease includes myocardial infarctions, peripheral artery disease, and any stroke. IQR, interquartile range; N/A, not applicable; NIHSS, National Institutes of Health Stroke Scale; NS, not significant; LDL, low-density lipoprotein; mRS, modified Rankin Scale.

**Table 2:** Linear regression for serum insulin-like growth factor I (s-IGF-I) per increase in pre-stroke physical activity (PA).

Type of s-IGF-I/Regression Model	B Per Increase in PA (B = ng/mL s-IGF-I)	*p*	N (PA1–4)	Partial Correlation
**Baseline s-IGF-I level**				
Model 1 [PA/A/S]	12.9 (2.52–21.9)	0.014	363	0.13
Model 2 [PA/A/S/history]	12.1 (2.34–21.8)	0.015	363	0.13
Model 3 [PA/A/S/history/B/C]	9.7 (−0.33–19.8)	0.058	356	0.10
Model 4 [PA/A/S/history/B/C/I]	9.7 (−0.38–19.7)	0.059	356	0.10
**Change in s-IGF-I level (ΔIGF-I)**				
Model 1 [PA/A/S]	8.9 (0.29–17.5)	0.043	334	0.11
Model 2 [PA/A/S/history]	9.3 (0.62–18.0)	0.036	334	0.12
Model 3 [PA/A/S/history/B/C]	9.2 (0.36–18.0)	0.041	332	0.11
Model 4 [PA/A/S/history/B/C/I]	9.7 (1.1–18.4)	0.028	332	0.12

Multiple linear regressions of the baseline and changes in s-IGF-I levels (ΔIGF-I) with respect to pre-stroke physical activity (PA). The unstandardized B values (s-IGF-I, ng/mL) with corresponding 95% confidence intervals (CI) per unit of PA are shown. In addition, the partial correlations for each of the models are given. Models 1–4 are shown with the following successively added adjustments for PA: age (A), sex (S), history of previous stroke and myocardial infarction (history), body mass index (B), traditional cardiovascular covariates (C, see Methods), and initial stroke severity (I).

## Supplementary Materials

Supplementary materials can be found at https://www.mdpi.com/2227-9059/8/3/52/s1.

## Abbreviations

BMI	Body mass index
IGF-I	Insulin-like growth factor I
NIHSS	National Institutes of Health Stroke Scale
PA	Physical activity
SGPALS	Saltin-Grimby Physical Activity Level
