# Supplementary material for: Relationship between Levels of Pre-Stroke Physical Activity and Post-Stroke Serum Insulin-Like Growth Factor I
Source: Biomedicines. 2020 Mar 4;8(3):52. doi: 10.3390/biomedicines8030052 (PMC7148508; doi:10.3390/biomedicines8030052)
Supplement: Supplementary file 1 [file biomedicines-08-00052-s001.pdf]

Front matter:

Title:

Relationship between levels of pre-stroke physical activity and post-stroke serum insulin-like growth factor I

Author list:

N. David Åberg [1, 2] *, Gustaf Gadd [1, 2], Daniel Åberg [1, 2], Peter Hällgren [2], Christian Blomstrand [3], Katarina Jood [3], Michael Nilsson [4, 5,], Frederick Rohan Walker [4, 5,], Johan Svensson [1], Christina Jern [6, 7], Jörgen Isgaard [1, 4, 5]

BACK MATTER:

Supplementary information:

Detailed description of the study populations and clinical examination (item number)

1. The Sahlgrenska Academy Study on Ischemic Stroke (SAHLSIS): The study population comprised adult Caucasian patients who presented with first-ever or recurrent ischemic stroke (IS) before reaching the age of 70 years (N=600). The patients were consecutively recruited between 1998 and 2003 at four stroke units in western Sweden. Caucasian population controls (N=600) were included as previously reported [1,2]. In the present study, a selection of patients with data available on pre-stroke physical activity and s-IGF-I levels is used (N=380). Blood samples were obtained in the acute phase, early (median of 4 days) and at 3 months (median of 101

days) after the index stroke [3]. Blood samples were drawn between 8.30 AM and 10.30 AM following overnight fasting. Serum was isolated within 2 h by centrifugation at 2000 × *g* at 4°C for 20 min, and stored at -80°C for 5–10 years before assay. Serum samples were analyzed for IGF-I in 2008 using a commercially available IGF-I binding protein (IGFBP)-blocked radioimmunoassay (RIA) (Mediagnost GmbH, Reutlingen, Germany) as described previously [3]. The intra-assay coefficient of variation (CV) was 5.1%, and the inter-assay CV was 4.7% in our sample range. The lower detection limit allowed precise measurement of very low IGF-I concentrations (0.09 ng/ml) according to the manufacturer, well below the present concentrations. Because s-IGF-I is considered to be very stable [4], we did not specifically test long-term stability in our study.

2. Clinical definitions and descriptions: Ischemic stroke (IS) was defined as an episode of focal neurological deficits with acute onset and lasting for >24 hours or until death, with no apparent non-vascular cause, and no signs of primary hemorrhage on brain imaging. The clinical rate of thrombolysis in 1998-2003 was very low, in this material only 1.3% (N=5), and there were no patients having undergone thrombectomy. All patients underwent ECG and neuroimaging with computed tomography (CT) and/or magnetic resonance imaging (MRI). Extracranial carotid and vertebral duplex ultrasound, MR angiography, catheter angiography, transcranial Doppler ultrasound, and transthoracic and/or transesophageal echo-cardiography were performed when clinically indicated. Both in the acute phase of stroke and 3 months post-stroke, all the patients were examined by a physician trained in stroke medicine. Information on the subjects' vascular risk factors was collected as described previously [1]. Hypertension was defined by pharmacological treatment for hypertension, systolic blood pressure

≥160 mm Hg, and/or diastolic blood pressure ≥90mm Hg. Diabetes mellitus was defined by diet or pharmacological treatment, fasting plasma glucose ≥7.0 mmol/L or fasting blood glucose ≥6.1 mmol/L. Smoking habits were coded as current, never or former. Concentrations of low-density lipoprotein (LDL, mmol/L) were measured as before [1,2]. The missing values (N=38, Table 1) were imputed using the mean of the baseline LDL levels to have complete LDL datasets when used as a covariate in the regression models.

3. Maximum stroke severity within the first 10 days after the stroke was scored using the Scandinavian Stroke Scale (SSS). SSS is a 58-point scale, in which a higher score represents better functionality. The SSS is highly (but inversely) correlated to the National Institutes of Health Stroke Scale (NIHSS). In this study, global SSS scores were transformed to NIHSS scores using a conversion algorithm [5], as described in the main text. Functional outcome 3 months and 2 years after IS was assessed according to the modified Rankin Scale (mRS) [6], later modified to seven steps (0-6) [7]. The mRS 0-2 scores represent functional independence, whereas scores 3-5 represent functional dependence, and mRS 6 designates death.
4. The Saltin-Grimby Physical Activity Level Scale (SGPALS) has four groups of self-reported physical activity (PA) levels. These are; PA1 (inactive group), which is defined as "almost completely inactive, reading, watching TV and movies, etc."; PA2 (active group), defined as "some physical activity during at least 4 hours per week: riding a bicycle or walking to work, walking or skiing with the family, gardening"; PA3 (intensive group), defined as "regular activity, such as heavy gardening, running, calisthenics, tennis, etc."; and PA4 (elite intensive group) defined as "regular

hard physical training for competition in running events, soccer, racing, European handball, etc. several times per week.” [8].

5. All participants provided informed consent prior to enrollment. For participants who were unable to communicate, consent was obtained from their next-of-kin. This study was approved by the Ethics Committee of the University of Gothenburg.

References

1. Jood, K.; Ladenvall, C.; Rosengren, A.; Blomstrand, C.; Jern, C. Family history in ischemic stroke before 70 years of age: the Sahlgrenska Academy Study on Ischemic Stroke. *Stroke* **2005**, *36*, 1383-1387, doi:10.1161/01.STR.0000169944.46025.09.
2. Olsson, S.; Jood, K.; Blomstrand, C.; Jern, C. Genetic variation on chromosome 9p21 shows association with the ischaemic stroke subtype large-vessel disease in a Swedish sample aged ≤ 70. *Eur J Neurol* **2011**, *18*, 365-367, doi:10.1111/j.1468-1331.2010.03096.x.
3. Åberg, D.; Jood, K.; Blomstrand, C.; Jern, C.; Nilsson, M.; Isgaard, J.; Aberg, N.D. Serum IGF-I levels correlate to improvement of functional outcome after ischemic stroke. *J Clin Endocrinol Metab* **2011**, *96*, E1055-1064, doi:10.1210/jc.2010-2802.
4. Burns, C.; Rigsby, P.; Moore, M.; Rafferty, B. The First International Standard For Insulin-like Growth Factor-1 (IGF-1) for immunoassay: preparation and calibration in an international collaborative study. *Growth hormone & IGF research : official journal of the Growth Hormone Research Society and the International IGF Research Society* **2009**, *19*, 457-462, doi:10.1016/j.ghir.2009.02.002.
5. Gray, L.J.; Ali, M.; Lyden, P.D.; Bath, P.M.; Collaboration, V.I.S.T.A. Interconversion of the National Institutes of Health Stroke Scale and Scandinavian Stroke Scale in acute stroke. *J Stroke Cerebrovasc Dis* **2009**, *18*, 466-468, doi:10.1016/j.jstrokecerebrovasdis.2009.02.003.
6. Rankin, J. Cerebral vascular accidents in patients over the age of 60. II. Prognosis. *Scott Med J* **1957**, *2*, 200-215, doi:10.1177/003693305700200504.
7. Banks, J.L.; Marotta, C.A. Outcomes validity and reliability of the modified Rankin scale: implications for stroke clinical trials: a literature review and synthesis. *Stroke* **2007**, *38*, 1091-1096, doi:10.1161/01.STR.0000258355.23810.c6.
8. Saltin, B.; Grimby, G. Physiological analysis of middle-aged and old former athletes. Comparison with still active athletes of the same ages. *Circulation* **1968**, *38*, 1104-1115.
